# Dermatomyositis and Polymyositis: Are Both Ends of a Same Spectrum?

**DOI:** 10.1111/jocd.70676

**Published:** 2026-01-16

**Authors:** Yashdeep Singh Pathania

**Affiliations:** ^1^ Department of Dermatology, Venereology and Leprology All India Institute of Medical Sciences, Rajkot Rajkot Gujarat India

**Keywords:** CADM, dermatomyositis, DM, PM, polymyositis

Dear Editor,

Idiopathic inflammatory myopathies (IIM), also commonly known as myositis, are a diverse cluster of autoimmune disorders with multifarious clinical presentations. IIM may further be classified into several subgroups, which include dermatomyositis (DM) (including hypo myopathic and amyopathic dermatomyositis), anti‐synthetase syndrome (ASyS), immune‐mediated necrotizing myopathy (IMNM), inclusion body myositis (IBM), polymyositis (PM), and overlap myositis [1]. Overlap myositis is the commonest type of IIM. Polymyositis (PM) is now considered rare after reclassification of many legacy cases to IMNM, ASyS, IBM, or overlap myositis. PM should be a diagnosis of exclusion.

As per the diagnostic criteria by Bohan and Peter, DM is categorized as one of the IIMs in which myositis is essential for the diagnosis [2]. The typical skin manifestations linked with symptomatic myositis are seen in classical DM. Formerly, DM was contemplated to constitute one end of the dermatomyositis/polymyositis (PM) spectrum with both skin and muscle involvement, albeit PM was the other extreme end limited to muscle only. This concept led to previous clinical trials, and epidemiologic studies to be grouped as one disease without clear distinction leading to intricate analyses. DM and PM are considered disparate entities now as both diseases have different underlying pathophysiological mechanisms, clinical features, and autoantibody profiles. The clinically amyopathic DM (CADM) is the skin predominant form of DM, including both the hypomyopathic and amyopathic DM (ADM). When patients do not show obvious clinical myositis but has minimum one abnormal muscle‐related investigation such as muscle imaging, biopsy, or related enzymes, it is considered hypomyopathic DM, whereas all investigations are within normal limits in ADM. Albeit, when typical and characteristic DM skin lesions exist for at least 6 months without history or clinical evidence of muscle weakness, it is known as CADM [3].

There has been misclassification in about 55.6% patients as per older concept where CADM was excluded from IIM classification leading to delay in exact diagnosis by approximately 15 months. The newer concept of CADM has gained attention through fields of neurology and rheumatology as European Neuromuscular Centre (ENMC) timely propose their proceedings relating to DM. The current systems of classification are vigorously attempting to include cutaneous DM features without the muscle involvement to classify patients as Amyopathic or skin predominant DM. A study by Patel et al. [4] found that these typical dermatological findings still missed 26.3% of ADM patients because the patients had other dermatological manifestations of DM.

In order to curtail the ambiguity and increase the accuracy, the signs or eponyms such as Gottron papules and Gottron sign over the dorsal metacarpophalangeal (MCP) or interphalangeal (IP) joints have been replaced with clinical descriptions such as scaly or non‐scaly, plane topped erythematous papules or plaques and macular erythema, respectively. The classification criteria also include serological investigations like DM‐specific myositis antibodies, and contributing factors of interstitial lung disease on computed tomography and muscle weakness despite skin symptoms. There are indications additional variables may be included in the criteria which may ameliorate the classification of CADM, and also integrate with the American College of Rheumatology (ACR) and the European League Against Rheumatism (EULAR) criteria which are unable to classify ADM and do not include myositis specific antibodies, albeit the criteria is undergoing additional scrutiny [5]. As DM is strongly associated with a higher risk of various internal malignancies (e.g., ovarian, lung, pancreatic), early detection of cases by classifying as skin predominant DM would mitigate the overall risk of mortality through appropriate screening for associated systemic conditions like interstitial lung disease (ILD) or malignancy, thereby strategizing the management plans. In conclusion, the current understanding of DM diagnosis is centered around including varied dermatological manifestations in the criteria for which various scientific agencies are in race to validate a more feasible consensus criteria for the diagnosis of DM.

## 1 Key Message

Dermatomyositis and polymyositis are not ends of the same spectrum. There are ongoing efforts for more skin focused and refined classification criteria for dermatomyositis.

## Author Contributions

Design and manuscript preparation and editing (Yashdeep Singh Pathania).

## Funding

The author has nothing to report.

## Conflicts of Interest

The author declares no conflicts of interest.

## Data Availability

Data sharing is not applicable to this article as no datasets were generated or analyzed during the current study.

## References

[1] I. E. Lundberg , M. Fujimoto , J. Vencovsky , et al., “Idiopathic Inflammatory Myopathies,” Nature Reviews. Disease Primers 7, no. 1 (2021): 86, 10.1038/s41572-021-00321-x. 34857798

[2] A. Bohan and J. B. Peter , “Polymyositis and Dermatomyositis (First of Two Parts),” New England Journal of Medicine 292 (1975): 344–347, 10.1056/NEJM197502132920706. 1090839

[3] H. J. Kim and V. P. Werth , “Updates in Dermatomyositis: Newer Treatment Options and Outcome Measures From Dermatologic Perspectives,” Annals of Dermatology 36, no. 5 (2024): 257–265, 10.5021/ad.24.022. 39343752 PMC11439981

[4] B. Patel , N. Khan , and V. P. Werth , “Applicability of EULAR/ACR Classification Criteria for Dermatomyositis to Amyopathic Disease,” Journal of the American Academy of Dermatology 79 (2018): 77–83. 29291954 10.1016/j.jaad.2017.12.055

[5] J. S. S. Concha , S. Pena , R. G. Gaffney , et al., “Developing Classification Criteria for Skin‐Predominant Dermatomyositis: The Delphi Process,” British Journal of Dermatology 182 (2020): 410–417, 10.1111/bjd.18096. 31049930

